# Deep-ocean origin of the freshwater eels

**DOI:** 10.1098/rsbl.2009.0989

**Published:** 2010-01-06

**Authors:** Jun G. Inoue, Masaki Miya, Michael J. Miller, Tetsuya Sado, Reinhold Hanel, Kiyotaka Hatooka, Jun Aoyama, Yuki Minegishi, Mutsumi Nishida, Katsumi Tsukamoto

**Affiliations:** 1 Ocean Research Institute, The University of Tokyo, Tokyo 164-8639, Japan; 2 Natural History Museum and Institute, Chiba, Chiba 266-8682, Japan; 3 Johann Heinrich von Thünen-Institut22767, Hamburg, Germany; 4 Osaka Museum of Natural History, Osaka 546-0034, Japan

**Keywords:** evolutionary origin, catadromy, mitogenome, migration

## Abstract

Of more than 800 species of eels of the order Anguilliformes, only freshwater eels (genus *Anguilla* with 16 species plus three subspecies) spend most of their lives in freshwater during their catadromous life cycle. Nevertheless, because their spawning areas are located offshore in the open ocean, they migrate back to their specific breeding places in the ocean, often located thousands of kilometres away. The evolutionary origin of such enigmatic behaviour, however, remains elusive because of the uncertain phylogenetic position of freshwater eels within the principally marine anguilliforms. Here, we show strong evidence for a deep oceanic origin of the freshwater eels, based on the phylogenetic analysis of whole mitochondrial genome sequences from 56 species representing all of the 19 anguilliform families. The freshwater eels occupy an apical position within the anguilliforms, forming a highly supported monophyletic group with various oceanic midwater eel species. Moreover, reconstruction of the growth habitats on the resulting tree unequivocally indicates an origination of the freshwater eels from the midwater of the deep ocean. This shows significant concordance with the recent collection of mature adults of the Japanese eel in the upper midwater of the Pacific, suggesting that they have retained their evolutionary origin as a behavioural trait in their spawning areas.

## 1. Introduction

**Figure 1.**
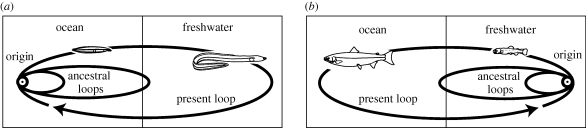
A diagram showing expanding ‘migration loops’ from the original spawning area (origin), which may have resulted in the establishment of the diadromous life histories of the (*a*) freshwater eels and (*b*) salmon (Tsukamoto *et al*. 2002).

The long spawning migrations of the catadromous anguillid eels from freshwater to far out in the ocean have fascinated scientists for almost a century (e.g. Tsukamoto 1992), but the evolutionary origins of this remarkable life history have remained hard to understand, as all other eels live in the ocean and do not make such impressive migrations (Miller & Tsukamoto 2004). As all other anguilliforms have totally marine life histories, and as tropical freshwater eels with short spawning migrations (Aoyama *et al*. 2003) occupy more basal positions in the published phylogenies, Tsukamoto *et al*. (2002) hypothesized two phases in the evolution of catadromous migrations of freshwater eels: (i) their migratory behaviour originated in tropical ocean areas and (ii) that tropical species subsequently expanded their ‘migration loops’ (representative migratory pathway of a species), and began to use fresh waters at higher latitudes for their growth (figure 1*a*). This resulted in the diversification of freshwater eels to include temperate species that make long migrations back to their tropical spawning areas. This argument is consistent with the Gross *et al*. (1988) prediction that catadromy has evolved in low-latitude tropical areas where productivity in freshwater exceeds that in the ocean. In contrast to eels, the freshwater origin of anadromous salmon has been demonstrated (Ishiguro *et al*. 2003), which indicates that they expanded their life histories to include the use of the ocean for growth while still returning to freshwater for reproduction (figure 1*b*).

The hypothesis of a sea water origin of the freshwater eels seems certain following a compelling argument by Tsukamoto *et al*. (2002). Nevertheless, the anguilliforms contain a diverse array of marine fishes, ranging from benthic shallow-water dwellers to deep-shelf, slope and abyssal plain inhabitants, as well as highly modified assemblages of open-water, meso- and bathypelagic species. Thus, the habitats used by marine eels provide no clues as to the origin of the freshwater eels. This question has been made even more difficult because morphological (Robins 1989) and molecular data (Inoue *et al*. 2004; López *et al*. 2007) have provided conflicting pictures of their higher level relationships, although the former study was merely based on a single, reductive character (fused versus divided frontal bones) and the latter studies had insufficient taxonomic representation to address questions regarding the evolutionary origin of the freshwater eels.

In this study, we conducted a phylogenetic analysis based on the whole mitogenome sequences of 58 species (including 31 newly determined sequences) representing all of the 19 families of the Anguilliformes (including the four ‘saccopharyngiform’ families), plus two outgroups, to address the evolutionary origin of the freshwater eels in a phylogenetic context. The resultant tree topology explicitly demonstrates that the freshwater eels form an exclusive clade with diverse oceanic midwater species placed in six families. This surprising discovery offers a new perspective on the evolutionary origin of the freshwater eels and may provide novel insights into the evolutionary process of their unique catadromous migrations.

## 2. Material and methods

We assembled whole mitogenome sequences from 56 anguilliforms representing all 15 anguilliform families, plus all four saccopharyngiform families currently recognized (Nelson 2006). Because the latter is nested within the former (Inoue *et al*. 2004), we refer to those eels of the two orders collectively as ‘anguilliforms’. We newly determined the whole mitogenome sequences from 31 anguilliform species using a combination of long and short polymerase chain reactions and direct cycle sequencing techniques following the methods developed by Miya & Nishida (1999). All sequence data are available from DDBJ/EMBL/GenBank (see the electronic supplementary material, table S1).

Mitogenome sequences from the 56 anguilliforms plus two outgroups were arranged into the typical gene order of vertebrates, aligned with MAFFT v. 6 (Katoh & Toh 2008), and then manually correcting them. The dataset comprises 10 821 positions from 12 protein-coding genes (excluding ND6 gene), 1728 positions from two rRNA genes and 1152 positions from 22 tRNA genes (13 701 total positions). For the third codon positions in the protein-coding genes, we arbitrarily used ‘A’ and ‘C’ instead of R and Y in RY coding to remove likely noise from quickly saturated transitional changes in the third codon positions, but maintaining phylogenetic signals by retaining all available positions in the dataset.

Unambiguously aligned sequences were divided into five partitions (first, second and third codon positions, rRNA, and tRNA genes) and the dataset was subjected to the partitioned maximum-likelihood (ML) analysis using RAxML v. 7.2.4 (Stamatakis 2006). The best-scoring ML tree was estimated using a general time reversible (GTR) + gamma (*Γ*) model of sequence evolution (the model recommended by the author) with 1000 bootstrap replicates (−f an option in RAxML).

According to the classification of growth habitats of anguilliform fishes of Miller & Tsukamoto (2004), the ancestral growth habitats in anguilliforms were reconstructed using ML and Bayesian approaches using Mesquite v. 2.6 (Maddison & Maddison 2009) and SIMMAP v.1.0 Beta 2.4 (Bollback 2006), respectively. Four discrete character states were assigned to the growth habitats of the terminal nodes: shallow water (character state 0), outer shelf and slope (state 1), oceanic midwater (state 2) and freshwater (state 3).

More details about methods can be found in the electronic supplementary material.

## 3. Results and discussion

**Figure 2.**
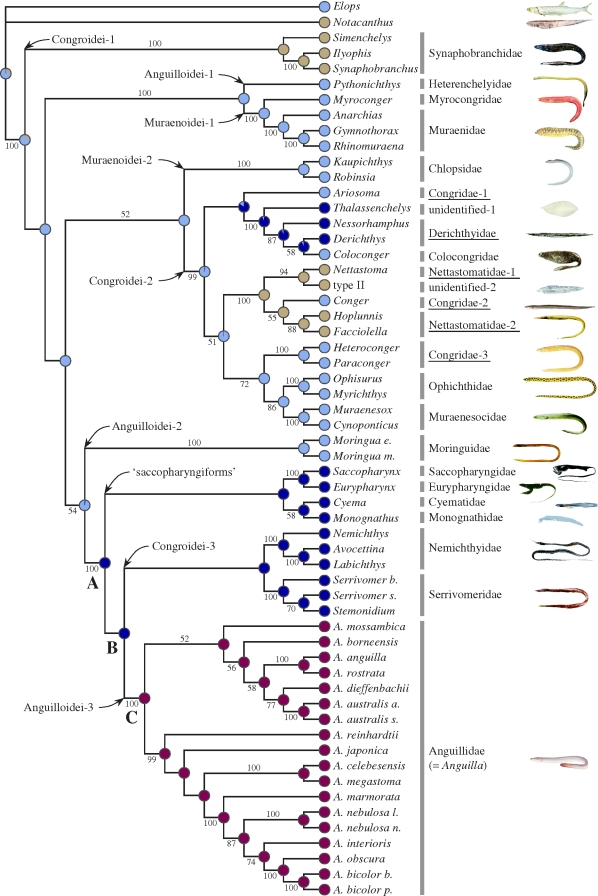
The best-scoring maximum likelihood (ML) tree of 58 elopomorph species based on unambiguously aligned whole mitogenome sequences (13 701 positions). Numerals beside internal branches indicate BPs of ≥50% based on 1000 replicates. Evolution of the adult growth habitats is reconstructed on the ML tree under an ML optimality criterion. A pie chart at each node indicates the likelihoods for these four character states. Scientific names of non-monophyletic families are underlined. Light blue circle, shallow water; brown circle, outer shelf and slope; dark blue circle, oceanic midwater; red circle, freshwater.

The partitioned ML analysis resulted in a relatively well-resolved tree, with approximately 70 per cent of the internal branches supported by moderate to high (70–100%) bootstrap probabilities (BPs; figure 2). Although the four families of the ‘saccopharyngiforms’ form a monophyletic group in the resultant tree, the clade is weakly supported (<50% BP) and is deeply nested within the anguilliforms as shown in the previous study (Inoue *et al*. 2004). All the three currently recognized suborders (Congroidei, Anguilloidei and Muraenoidei) are recovered as polyphyletic, with two or three unnested monophyletic groups recognized for each suborder (e.g. Anguilloidei clades 1, 2, 3 in figure 2). Of the 12 families, for which we sampled two or more taxa, nine are confidently recovered as monophyletic with 100 per cent BPs. The rest of the three congroid families (Derichthyidae, Nettastomatidae and Congridae), on the other hand, are recovered as either para- or polyphyletic within a more comprehensive clade (Congroidei-2) supported by 100 per cent BP (figure 2).

Apparently, the higher level classification of the anguilliforms requires substantial revision based on more extensive taxon and character sampling. Significantly, however, the present ML tree unequivocally places the freshwater eels at the top of the anguilliform phylogenies and they are nested within a more comprehensive monophyletic group (clade A) supported by 100 per cent BP. Other than the freshwater eels, clade A comprised the four saccopharyngiform families (Saccopharyngidae, Eurypharyngidae, Cyematidae and Monognathidae) and two congroid families (Nemichthyidae and Serrivomeridae), with these six families containing 47 species placed in 10 genera (Nelson 2006). Interestingly, these 47 species are all oceanic midwater dwellers, occurring mainly at tropical and subtropical meso- and bathypelagic depths (200–3000 m) throughout their adult stages with no exception (Miller & Tsukamoto 2004). As expected from the resultant phylogenies, the ML and Bayesian reconstruction of the ancestral growth habitats explicitly indicate that the freshwater eels evolved from an oceanic midwater ancestor (figure 2), with the character state 2 (oceanic midwater) being most likely at nodes A and B (*p* > 0.99).

How can we explain why this apparent evolutionary shift of the freshwater eel life history from the oceanic midwater to freshwater occurred? These two environments are remarkably different and require fishes to be adapted to very different ecological and physiological constraints. One possibility is that in tropical regions at the time of the divergence of anguillids, there was a productivity gradient between freshwater and marine environments as hypothesized by Gross *et al*. (1988), which made it advantageous for feeding success to invade freshwater. The importance of this relationship is supported by apparent clines in freshwater use in temperate anguillid species, with fewer eels entering freshwater at the northern margins of their ranges where productivity is much lower than in the estuarine and coastal habitats (Tsukamoto *et al*. 1998, 2009). Another possibility is that because there were probably no eels in freshwater at that time, compared with the presence of multiple lineages of eels in marine environments (figure 2), including voracious predators such as moray eels (family Muraenidae), there was a vacant niche for eels in freshwater. In addition to the lack of competition with other eels, most freshwater habitats also might have had fewer large predators that could prey on eels.

Regarding the characteristics of the first freshwater eels, it should be noted that the present ancestral character reconstruction is based on the traits of the extant species and thus it does not specify the character state between nodes B and C (figure 2). If there was an ancestral eel lineage between these two nodes that went extinct long ago and lived at shallower depths than present-day mesopelagic eels, then the habitat shift into freshwater would have been more gradual. This ancestor may have eventually come to estuaries during their larval or juvenile phases and developed an adaptive behaviour of regularly inhabiting estuaries and occasionally entering freshwater in tropical regions because of higher food availability, better survival or to escape from predators (Tsukamoto *et al*. 2009). Once natural selection resulted in the emergence of eels that regularly used freshwater for growth, a new catadromous life history was established, with the eels still using the open ocean as their spawning area.

The closest relatives of anguillid eels found in this study are the mesopelagic eels of the Nemichthyidae and Serrivomeridae that spawn in the open ocean, with their larvae mixing in the ocean surface layer with those of anguillids (Miller *et al*. 2006). The recent capture of mature adults of the Japanese eel, *Anguilla japonica*, at depths of about 220–280 m in the western North Pacific (Chow *et al*. 2009), shows that anguillids have retained the apparent ancestral trait of offshore pelagic spawning. Reproductive behaviour is typically conservative and constrained by many ecological and physiological factors (Tsukamoto *et al*. 2002), so the catadromous migration of freshwater eels back to their offshore habitats over an evolutionary time scale represents a remarkable relic of the reproductive behaviour of these enigmatic animals that share a common ancestry with pelagic eels of the deep ocean.
